# The Measurement of Individual Differences in Cognitive Biases: A Review and Improvement

**DOI:** 10.3389/fpsyg.2021.630177

**Published:** 2021-02-18

**Authors:** Vincent Berthet

**Affiliations:** ^1^Université de Lorraine, 2LPN, F-54000 Nancy, France; ^2^Centre d'Économie de la Sorbonne, CNRS UMR 8174, Paris, France

**Keywords:** cognitive biases, measurement, individual differences, judgment and decision making, decision biases

## Abstract

Individual differences have been neglected in decision-making research on heuristics and cognitive biases. Addressing that issue requires having reliable measures. The author first reviewed the research on the measurement of individual differences in cognitive biases. While reliable measures of a dozen biases are currently available, our review revealed that some measures require improvement and measures of other key biases are still lacking (e.g., confirmation bias). We then conducted empirical work showing that adjustments produced a significant improvement of some measures and that confirmation bias can be reliably measured. Overall, our review and findings highlight that the measurement of individual differences in cognitive biases is still in its infancy. In particular, we suggest that contextualized (in addition to generic) measures need to be improved or developed.

## Introduction

Since the seminal work of Kahneman and Tversky on judgment and decision-making in the 1970s, there has been a growing interest for how human judgment violates normative standards (e.g., Tversky and Kahneman, 1974; Kahneman et al., 1982; Gilovich et al., 2002). When making judgments or decisions, people often rely on simplified information processing strategies called heuristics, which may lead to systematic—and therefore predictable—errors called *cognitive biases* (hereafter CB). For instance, people tend to overestimate the accuracy of their judgements (overconfidence bias), to perceive events as being more predictable once they have occurred (hindsight bias), or to carry on fruitless endeavors in which they already have invested money, time or effort (sunk cost fallacy). Although the “heuristics and biases” program has raised criticism regarding its pessimistic view human rationality and its lack of theoretical ground, it has been remarkably fruitful. To date, behavioral scientists have identified dozens of CB and heuristics that affect judgment and decision-making significantly (e.g., Baron, 2008, listed 53 such biases) and have proposed several taxonomies (e.g., Carter et al., 2007; Stanovich, 2009; Pohl, 2017).

The “heuristics and biases” program led to a large body of research investigating how these mental shortcuts may impede decision-making in areas such as management (e.g., Maule and Hodgkinson, 2002), medicine (e.g., Blumenthal-Barby and Krieger, 2015), law (e.g., Rachlinski, 2018), or finance (e.g., Baker and Nofsinger, 2002). However, individual differences have been largely neglected in this endeavor (Stanovich et al., 2011; Mohammed and Schwall, 2012). In fact, most of the current knowledge about the impact of CB on decision-making relies upon experimental research and group comparisons (Gilovich et al., 2002), which might lead to the false inference that every single individual is susceptible to CB, and to the same extent.

Still, there has been a growing interest in going beyond aggregate level results by examining individual differences (e.g., Stanovich and West, 1998, 2000). This line of research has led to two noteworthy findings. The first one is that performance on CB tasks is only moderately correlated to cognitive ability, which suggests that a major part of the reliable variance of scores on CB tasks is unique (e.g., Stanovich and West, 2008; Stanovich, 2012; Teovanović et al., 2015; Bruine de Bruin et al., 2020). The second finding is that correlations between CB measures are low, suggesting the absence of any general factor of susceptibility to CB. Indeed, exploratory factor analysis reveals that at least two latent factors can be extracted from the intercorrelations between the scores on various CB tasks (Parker and Fischhoff, 2005; Bruine de Bruin et al., 2007; Aczel et al., 2015; Teovanović et al., 2015).

### The Measurement of Cognitive Biases: A Review

It is worth noting that research on individual differences in CB has been conducted despite a lack of psychometrically sound measures^1^. Here, we review this research topic in order to inventory which reliable measures are currently available. Note that self-report measures have been developed to assess the propensity to exhibit biases such as the bias blind spot (Scopelliti et al., 2015), correspondence bias (Scopelliti et al., 2018), and confirmation bias (Rassin, 2008). In this review, we considered only objective measures of individual differences in CB (i.e., based on performance on experimental tasks).

The development of reliable measures of CB faces several challenges. As a preliminary point, one should distinguish between two types of CB tasks. Some CB are measured by a single or a few equivalent items. For example, the gambler's fallacy can be assessed with a single problem such as “When playing slot machines, people win something about 1 in every 10 times. Julie, however, has just won on her first three plays. What are her chances of winning the next time she plays?” (Toplak et al., 2011). Likewise, base rate neglect, sunk cost fallacy, and belief bias are usually measured by a single or several equivalent items. For those biases, bias susceptibility is measured with respect to accuracy and the measurement of individual differences raises no particular methodological issue.

Other CB are evidenced by the effect of a normatively irrelevant factor on judgments or decisions, which is typically manipulated between subjects. For example, the framing effect is usually obtained by presenting a gain and a loss version of a same decision problem to two different groups (e.g., Tversky and Kahneman, 1981). Between-subjects designs are also used for anchoring bias, hindsight bias, and outcome bias. Therefore, a first challenge in the measurement of CB is to adapt between-subjects designs to within-subjects ones. In the latter case, bias susceptibility is measured by comparing each subject's responses to the different conditions. For example, the framing effect is also found using a within-subjects design (Frisch, 1993) where the two versions of the problem are separated in the questionnaire to avoid any memory effects (e.g., Parker and Fischhoff, 2005). Although there may be some limitations, the framing effect, anchoring bias, hindsight bias, and outcome bias can all be successfully assessed using within-subjects designs (Stanovich and West, 1998; Lambdin and Shaffer, 2009; Aczel et al., 2015).

A second challenge in the measurement of CB is to build reliable scores. Most studies that investigated individual differences in CB relied on composite scores derived from a large set of CB tasks (e.g., Toplak et al., 2011; Aczel et al., 2015). It turns out that such composite scores are unreliable (West et al., 2008; Toplak et al., 2011; Aczel et al., 2015). For instance, Toplak et al. (2011) reported that the internal consistency of composite scores consisting in the average performance on 15 classic heuristics and biases tasks was 0.484 (Cronbach's alpha). Likewise, Aczel et al. (2015) showed that the reliability of composite scores calculated as the sum of the scores (1 or 0) to 13 CB tasks was 0.37 for one form of the test and 0.23 for another parallel form (Cronbach's alpha). Even composite scores derived from various tasks measuring the same CB turned out to be unreliable (e.g., Rassin, 2008, in the case of confirmation bias). These studies, however, used a single item for each task, which is detrimental to score reliability. Moreover, such a practice affects the comparability of parallel versions of the same task (Aczel et al., 2015). On the other hand, using multiple items for each task allows for assessing the reliability of test scores, so that reliable scores can be aggregated irrespective to the format of the tasks from which they are derived (the same way as IQ scores result from aggregating scores to different subtests).

Two noteworthy studies sought to adjust CB tasks to improve scale reliability. Bruine de Bruin et al. (2007) evaluated the reliability and validity of a set of seven behavioral tasks (forming the Adult Decision-Making Competence; A-DMC) measuring different aspects of decision-making (resistance to framing, recognizing social norms, under/overconfidence, applying decision rules, consistency in risk perception, resistance to sunk costs, path independence). These authors adapted tasks from the Youth Decision-Making Competence (Y-DMC; Parker and Fischhoff, 2005) to achieve increased reliability with adults. For example, Parker and Fischhoff (2005) found relatively low internal consistency for the task measuring susceptibility to framing. To address that issue, Bruine de Bruin et al. increased the number of items and replaced the dichotomous choice by a 6-point rating scale (each endpoint reflecting a strong preference for one of the two original choice options). Bruine de Bruin et al. reported values of Cronbach's alpha ranging from 0.54 to 0.77 over the seven scales, and test-retest values around 0.50. Moreover, A-DMC scores showed evidence of criterion validity as they predicted the likelihood of reporting negative life events indicative of poor decision making.

In a similar vein, Teovanović et al. (2015) used multi-item tasks for the measurement of individual differences in seven CB (anchoring effect, belief bias, overconfidence bias, hindsight bias, base rate neglect, sunk cost effect, outcome bias). Here too, Teovanović et al. introduced adjustments to increase score reliability (especially for anchoring bias, see below). With the exception of hindsight bias, scores on all CB tasks reached satisfactory levels of reliability (Cronbach's alphas >0.70). This work represents a significant step forward in the measurement of individual differences in CB.

Finally, the unpublished work of Gertner et al. (2016) should be highlighted as a valuable attempt to develop a standardized assessment of CB in judgment and decision-making. These authors relied on a sound psychometric approach that started with identifying the facets of each bias to cover the most of each bias's construct. Accordingly, Gertner et al. used various tasks to measure each CB (e.g., the measurement of confirmation bias involves the Wason task, a task related to information search, and a task related to evaluation/weighting of evidence). While reporting acceptably high values of internal consistency for the different scales (with the exception of the confirmation bias scales), the test of Gertner et al. remained at an exploratory stage, calling for further development. As outlined by the authors, “the study of bias within an individual difference framework is still largely in its infancy” (p. 3).

## Aim of the Study

Taken together, the studies of Bruine de Bruin et al. (2007) and Teovanović et al. (2015) provide evidence that a set of eight CB can be reliably measured: framing, anchoring, belief bias, overconfidence, hindsight bias, base rate neglect, sunk cost fallacy, and outcome bias^2^. As the correlations between CB measures have been found to be low, this set may be viewed as an inventory of independent measures that could be used each separately. Such an inventory opens up a promising avenue to research on CB based on an individual differences approach. For example, the A-DMC (Bruine de Bruin et al., 2007) has been used to investigate executive functions in decision-making (Del Missier et al., 2010), age-related changes in decision-making competence (Del Missier et al., 2020b), and decision-making in schizophrenia (Del Missier et al., 2020a). However, this inventory should be both improved and extended. On the one hand, some measures are still inconvenient and therefore need to be improved. For instance, the measurement of outcome bias as reported by Teovanović et al. (2015) involves a 1-week delay between the two outcome conditions. On the other hand, reliable, multi-item, measures of key CB such as confirmation bias and availability bias are still lacking. The general aim of the study is to address those two issues by (1) replicating and improving the eight measures of CB identified, (2) testing a measure of confirmation bias. Open Science Practices: All data files are available at: https://osf.io/wfums/.

### Study 1

#### Method

The aim of study 1 was primarily to replicate the findings relative to the eight measures of CB identified using fewer items for each task. In fact, the combined use of these eight measures with their current number of items would result in long completion times. We investigated to what extent this item reduction would impact the reliability of the measures. In addition, we made several adjustments: the measurement procedure of the outcome bias was changed as compared to Teovanović et al. (2015) in such a way to obtain the measure in one setting (without a 1-week delay), and the scoring method for some measures (framing bias and hindsight bias) was fine-tuned. Items were drawn from three sources: the original measure, the existing literature, or they were new. The only criteria for including or not items from the original measure or the existing literature was whether they were suited for French participants. When the number of suitable items was not sufficient, new items adapted to that population were created. All items can be found in the Supplementary Material.

##### Participants

The participants were 163 unpaid undergraduate students (26 males, 137 females) who attended first-year introductory course in differential psychology at the University of Lorraine (France). Their mean age was 18.52 (*SD* = 1.89). Participants gave their informed consent before taking part in the study.

##### Measures

###### Framing Bias.

Framing is the tendency of people to be affected by how information is presented (Kahneman and Tversky, 1984). Based on the procedure reported by Bruine de Bruin et al. (2007), we measured a risky-choice framing effect (note that these authors also measured an attribute framing effect, using seven items for each framing task). Decision problems were presented to the subjects who chose between a sure-thing option (A) and a risky-choice option (B). Participants responded on a 6-point scale ranging from 1 (“I would definitely choose option A”) to 6 (“I would definitely choose option B”). Each decision problem had two versions, a gain version and a loss version. The two versions were identical, only the framing differed (e.g., Tversky and Kahneman, 1981; Fischhoff, 1983). Four decision problems (eight frames) were used, referring to various cases: an unusual disease (Tversky and Kahneman, 1981), a raise of income tax (Highhouse and Paese, 1996), selling an apartment (Fagley and Miller, 1997), and food poisoning in an African village (Svenson and Benson, 1993). Two of these decision problems are used in Bruine de Bruin et al. (2007). In Bruine de Bruin et al. (2007, p. 942), the framing bias is measured as “the mean absolute difference between ratings for the loss and the gain versions of each item” (accordingly, the scores range potentially from 0 to 5). However, prospect theory predicts a particular direction of risky-choice framing effects, subjects being more prone to choose the risky option in loss frames and the sure option in gain frames (Kahneman and Tversky, 1979). Therefore, we argue that framing scores should be calculated as the difference (rather than the absolute difference) between the mean ratings of the loss frames and the mean ratings of the gain frames. The gain and loss items appeared in separate blocks, with different item orders in each block (LeBoeuf and Shafir, 2003).

###### Hindsight Bias.

Hindsight bias is the tendency to overestimate *ex post* the likelihood of an outcome (Fischhoff, 1975). We used the procedure reported by Teovanović et al. (2015), which is based on a memory/recall design. In a first phase, participants performed a task in which they were asked to find the exception in a set of four words (e.g., “November,” “August,” “December,” and “January”) and then indicate the confidence in their response using a 5-point scale (the set of words used were new). Later in the test, participants received feedback on the accuracy of each response and were asked to recall their initial confidence judgment. Teovanović et al. (2015) calculated the hindsight score as the proportion of hindsighted responses (a response was coded as hindsighted if the participant lowered her confidence after being informed that her response was incorrect, or raised her confidence after being informed that her response was correct). However, such a scoring procedure does not consider the magnitude of the hindsight bias. Therefore, the difference between the confidence rating recalled and the initial one should be considered. Moreover, there is a hypothesized direction for this difference: it should be positive when a correct feedback is provided, and negative when an incorrect feedback is provided. Accordingly, the hindsight score was calculated as (recalled confidence rating—initial confidence rating) × accuracy, with accuracy being coded 1 (correct feedback) or −1 (incorrect feedback) (we thank an anonymous reviewer for suggesting this scoring method). We used fewer items than Teovanović et al. (10 vs. 14). As subjects rated their confidence on a 5-point scale, the potential range of scores was 0–40.

###### Overconfidence Bias.

Overconfidence has several aspects (Moore and Schatz, 2017) but it commonly refers to the tendency to overestimate one's own abilities. We used the standard measurement procedure in which participants respond to a performance task and then indicate the confidence in their response (e.g., Lichtenstein and Fischhoff, 1977). As Bruine de Bruin et al. (2007), we used dichotomous general knowledge items for the performance task. We used new items which were drawn from various tests used for the purpose of admission to competitions organized within the French civil service. Overconfidence was assessed through a calibration measure, defined as the difference between the mean confidence ratings and the mean accuracy (percentage of correct answers). Participants rated their confidence on a 6-point scale ranging from 50% (“I am just guessing”) to 6 (“I absolutely sure”). Therefore, scores ranged from −50 (maximum underconfidence) to 100 (maximum overconfidence). We used fewer items than Bruine de Bruin et al. (25 vs. 34).

###### Anchoring Bias.

Anchoring bias is the tendency of people to adjust their—numerical—judgments toward the first piece of information (Tversky and Kahneman, 1974). We used the procedure reported by Teovanović et al. (2015) who proposed to measure the anchoring bias as the difference between a numerical estimate following an anchor value and an initial, anchor-free, estimate made before the anchor presentation. Participants were first required to make numerical estimates relative to general knowledge (E1) (e.g., the average number of babies born per day in France). In a second phase, they were presented with the same set of items and performed a comparative task and a final estimation task. In the former, participants indicated whether the number to estimate was higher or lower than a given value (anchor, A). Anchor values were set automatically by multiplying anchor-free estimates (E1) with predetermined values (ranging from 0.2 to 1.8 between items). Then, participants provided their final estimate (E2). In each item, the anchoring bias was calculated as (E1 – E2)/(A – E1). Anchoring values lower than 0 (lack of anchoring) or higher than 1 (total anchoring) were removed (12.57% of all observations). The anchoring score is the average anchoring bias across items. Items were selected from the existing literature on anchoring (e.g., Jacowitz and Kahneman, 1995) and were very similar to that used by Teovanović et al. (2015). We used fewer items than these authors (12 vs. 24).

###### Outcome Bias.

Outcome bias is the tendency to evaluate the quality of a decision based on its outcome. This bias is typically evidenced in experiments where subjects are presented with a scenario describing a decision made by an individual (e.g., a physician who decided to go ahead with an operation.). In one condition, subjects are informed that the decision led to a positive outcome (e.g., “The operation succeeded”) and in another condition, subjects are informed that the decision led to a negative outcome (e.g., “The patient died”). Participants are asked to evaluate the quality of the decision itself. At the cost of reducing the effect size, outcome bias can be obtained in within-subjects designs (Baron and Hershey, 1988). Teovanović et al. (2015) reported a reliable measurement of the outcome bias using a within-subjects design in which subjects evaluate 10 decisions a first time and then a second time a week later but with different outcomes. Bias susceptibility amounts to the inconsistency between the responses to the two outcome conditions. However, the 1-week delay makes this procedure quite inconvenient. To address that issue, we used different items for the two outcome conditions at the cost of potentially increasing measurement error. To avoid confounding the effects of quality and outcome of the decision (a threat to construct validity), we chose a conservative approach by which decisions with a positive outcome were quite bad with respect to decision quality (e.g., “Celine was due to take an important college exam. Two days before, she was invited to a party. She decided to go. She had a great time and stayed with her friends until the early hours of the morning. The next day, she revised most of the day. She passed her exam”) while decisions with a negative outcome were quite good (e.g., “Paul was late for a college exam. Being stuck in traffic, he decided to walk to college as quickly as possible. But he arrived late and was not allowed to take the exam”). The bias score is defined as the difference between the mean ratings of decisions with positive outcomes and the mean ratings of decisions with negative outcomes. Five items were used per condition and participants were asked to rate the decision quality on a 6-point scale ranging from 1 (“It was a poor decision”) to 6 (“It was an excellent decision”). Seven items were selected from the existing literature (Baron and Hershey, 1988; Gino et al., 2009; Aczel et al., 2015; Teovanović et al., 2015) and three new items were created.

*Base Rate Neglect*. Base rate neglect is a bias in which the information regarding a specific case outweighs the information relative to prior probabilities (Bar-Hillel, 1980). On each item, participants were presented with two kinds of information: base-rates (e.g., “1,000 people participated in a study, including 4 men and 996 women”) and information concerning a specific case (e.g., “Dominique is a randomly chosen participant of this study. Dominique is 23 years old and is finishing a degree in engineering. On Friday nights, Dominique likes to go out cruising with friends while listening to loud music and drinking beer”). Participants were required to estimate a probability related to the specific case (“What is the probability that Dominique is a man?”) (free estimate). The bias score was defined as the proportion of responses that differed from the base rate information in the direction implied by the specific case (e.g., higher than 0.4% in the above example). In typical base-rate problems, the description of the specific case fits common stereotypes of the smaller population group, so that the description of the person and of the base rate are incongruent (De Neys and Glumicic, 2008). We used four such items, two of which were selected from De Neys and Glumicic (2008) and two were created.

*Sunk Cost Fallacy*. Sunk cost fallacy is the tendency to carry on fruitless endeavor because of the money, time or effort already invested (Arkes and Blumer, 1985). We used the same measurement procedure as Bruine de Bruin et al. (2007) and Teovanović et al. (2015). Participants were presented with hypothetical scenarios and choose between the sunk-cost option and the normatively correct option using a 6-point scale ranging from 1 (the normatively correct option) to 6 (the sunk-cost option). The bias score was defined as the mean rating score. We used fewer items than the two above studies (5 vs. 10 and 8, respectively). Three items were drawn from the existing literature (Arkes and Blumer, 1985; Bornstein and Chapman, 1995; Teovanović et al., 2015) and two were created.

*Belief Bias*. Belief bias is the tendency to evaluate deductive arguments based on the believability of the conclusion rather than its logical validity (Evans et al., 1983). We used the measurement procedure reported by Teovanović et al. (2015). Subjects were instructed to evaluate syllogisms by indicating whether the conclusion necessarily followed from the premises or not, assuming that all premises were true. The rationale is to assess the effect of the believability of the conclusion (believable vs. unbelievable) for a given level of validity of the argument (valid vs. invalid). Four pairs of syllogisms were used, each pair involving a consistent item and an inconsistent one. On four inconsistent items, the logical validity of the argument was incongruent with the believability of the conclusion (two of them were valid but unbelievable, and two were invalid but believable). On four consistent items, the logical validity of the argument was congruent with the believability of the conclusion (two of them were both valid and believable, and two were both invalid and unbelievable). The bias score was the number of biased responses. A response was coded as biased if the subject provided an incorrect answer to an inconsistent item and a correct answer to the corresponding consistent item. We used four pairs of syllogisms drawn from Teovanović et al. (2015).

##### Procedure

After providing consent, participants completed the eight tasks in the following order: (1) gain version items of the framing task, (2) the first phase of the hindsight task, (3) overconfidence bias, (4) anchoring bias, (5) outcome bias, (6) base rate neglect, (7) sunk cost fallacy, (8) belief bias, (9) the second phase of the hindsight task (recall), (10) loss version items of the framing task. After completing the test, participants were given feedback on the study.

#### Results

The mean testing time was 49.26 min (*SD* = 15.57). Six participants (3.68%) were excluded from the analysis because of abnormally long times to complete the test (superior to 2 *SD*), which resulted in a final sample of 157 participants. We reviewed the discriminative ability and reliability of the CB measures (see Table 1 for a summary of the results). With the exception of framing bias and outcome bias, medium to large effect sizes were found, with Cohen's *d* values ranging from 0.68 (overconfidence bias) to 2.76 (sunk cost fallacy).

**Table 1:** Descriptive statistics, discriminative properties and internal consistency of CB measures.

**Cognitive bias**	**Descriptive statistics**		**Cohen's d**	**Range**		**Normality deviations**		**Shapiro-Wilk test**		**Reliability**	
	**Mean**	**Standard deviation**		**Potential**	**Observed**	**Skewness**	**Kurtosis**	**W**	***p***	**Number of items**	**Internal consistency**
**Study 1 (*****N*** **= 157)**											
Framing bias	0.22	0.75	0.23	−5 to 5	−1.75 to 2.5	0.54	1.3	0.95	<0.001	4	0.15 (alpha)
Hindsight bias	3.38	3.62	0.93	−40 to 40	−4 to 15	0.71	0.28	0.96	<0.001	10	0.45 (alpha)
Overconfidence bias	7.22	10.69	0.68	−50 to 100	−22 to 34	0	0.02	0.99	0.39	25	0.36 (split-half)
Anchoring bias	0.13	0.12	1.05	0 to 1	0 to 0.57	1.25	1.53	0.88	<0.001	12	0.68 (split-half)
Outcome bias	0.11	1.24	0.14	−5 to 5	−2.6 to 5	0.52	1.2	0.98	<0.01	10	0.57 (split-half)
Base rate neglect	0.53	0.35	1.49	0 to 1	0 to 1	−0.14	−1.19	0.88	<0.001	4	0.70 (alpha)
Sunk cost fallacy	3.73	0.99	2.76	1 to 6	1 to 6	−0.27	−0.04	0.99	0.09	5	0.35 (alpha)
Belief bias	1.5	0.71	2.10	0 to 4	0 to 3	−0.73	−0.3	0.77	<0.001	4	−0.15 (alpha)
**Study 2 (*****N*** **= 104)**											
Framing bias	0.94	1.02	1.05	−5 to 5	−1.5 to 4.88	0.71	1.19	0.97	0.01	8	0.74 (alpha)
Overconfidence bias	6.1	15.43	0.39	−100 to 100	−34 to 66	0.68	1.62	0.97	0.02	25	0.81 (split-half)
Outcome bias	0.77	1.25	0.95	−5 to 5	−1.5 to 4.17	0.58	−0.05	0.96	<0.01	24	0.85 (split-half)
**Study 3 (*****N*** **= 102)**											
Hindsight bias	4.19	5.77	0.73	−60 to 60	−16 to 26	0.72	3.46	0.92	<0.001	15	0.62 (alpha)
Sunk cost fallacy	3.61	0.87	2.99	1 to 6	1 to 6	0.14	0.7	0.97	0.02	5	0.08 (alpha)
Confirmation bias	51.67	14.21	3.64	0 to 100	20.83 to 87.5	0.24	−0.19	0.99	0.47	4	0.68 (alpha)

*In Study 2, the results reported for overconfidence bias are those obtained when the task includes the Matrix Reasoning and Verbal Reasoning items*.

Regarding score reliability, Study 1 revealed three main findings. First, we failed to replicate four measures in particular. The measure of framing bias produced a small effect size (*M* = 0.22, Cohen's *d* = 0.23) and was unreliable (Cronbach's alpha = 0.15). Note that the scoring method of Bruine de Bruin et al. (2007) produced a greater internal consistency (0.50). The results regarding overconfidence bias were also below those reported by Bruine de Bruin et al. The mean overconfidence score was 7.22% and the internal consistency was 0.36. This low reliability was due to the fact that accuracy scores were themselves unreliable (split-half = 0.25) (on the contrary, confidence scores were reliable, split-half = 0.78). In fact, it is not surprising that scores to a general knowledge test show poor reliability given the diversity of the items. Sunk cost fallacy scores were unreliable (Cronbach's alpha = 0.35) despite mean and effect size values similar to those reported by Bruine de Bruin et al. (2007) and Teovanović et al. (2015) (*M* = 3.73, Cohen's *d* = 2.76). Belief bias scores were also unreliable (Cronbach's alpha = −0.15) despite an effect size similar to that reported by Teovanović et al. (Cohen's *d* = 2.10).

Second, the internal consistency of hindsight bias and outcome bias measures was quite poor too (0.45 and 0.57, respectively) but such values could be attributed to the low number of items used for each (10). When using the scoring method of Teovanović et al. (2015), the internal consistency of the hindsight bias measure was 0.48, a value below that reported by these authors (0.66). Third and finally, two measures reached quite acceptable levels of reliability. The internal consistency of the anchoring bias measure was below that reported by Teovanović et al. (2015) (0.68 vs. 0.77) but that difference could be attributed to the difference in the number of items used (12 vs. 24). Our value suggests, however, that a reliable measure of this bias can be achieved with <24 items. The internal consistency of the base rate neglect measure was acceptable (Cronbach's alpha = 0.70) despite a reduced number of items (4 vs. 10 in Teovanović et al.).

Table 2 shows the bivariate correlations between CB measures. Correlations were low (all *r* < 0.22), and only six were statistically significant. This finding confirms what has been found in previous studies. Hindsight bias had the higher number (3) of significant correlations (with anchoring, outcome bias and belief bias).

**Table 2:** Correlations between CB measures.

**Study 1 (*****N*** **= 157)**								
**Cognitive bias**	**1**	**2**	**3**	**4**	**5**	**6**	**7**	**8**
1. Framing bias								
2. Hindsight bias	−0.07							
3. Overconfidence bias	0.17^*^	0.09						
4. Anchoring bias	0.05	0.22^**^	−0.06					
5. Outcome bias	−0.04	0.22^**^	−0.01	0.03				
6. Base rate neglect	−0.19^*^	0.09	0.10	0.13	0.11			
7. Sunk cost fallacy	−0.03	0.04	0.03	0.04	0.04	−0.06		
8. Belief bias	−0.15	0.16^*^	−0.08	0.10	0.16^*^	0.12	−0.14	
**Study 2 (*****N*** **= 104)**								
**Cognitive bias**	**1**			**2**			**3**	
1. Framing bias								
2. Overconfidence bias	0.06							
3. Outcome bias	−0.10			0.08				
**Study 3 (*****N*** **= 102)**								
**Cognitive bias**	**1**			**2**			**3**	
1. Hindsight bias								
2. Sunk cost fallacy	−0.02							
3. Confirmation bias	−0.16			−0.07				

* *p < 0.05*,

** *p < 0.01, two-tailed*.

We performed factor analysis to investigate the factorial structure of the eight CB measures. While the Bartlett's test of sphericity was significant [χ^2^_(28)_ = 53.5, *p* < 0.01], the Kaiser-Meyer-Olkin measure for sampling adequacy (*KMO* = 0.49) suggested that the data were not suited for factor analysis. However, as the KMO value was just below the recommended minimum value of 0.5 (Kaiser, 1974), we still performed the analysis. A two-factor model with oblimin rotation was retained on the basis on previous findings (Bruine de Bruin et al., 2007; Teovanović et al., 2015). The two factors accounted for only 22% of the total variance. This finding (which is not surprising given the low correlations between CB measures) is very similar to that reported by Teovanović et al. (2015). Only framing bias loaded on the first factor while hindsight, anchoring, outcome and belief bias had loadings of at least 0.30 on the second factor (Table 3). The two factors were barely correlated (*r* = −0.16). Note that these findings should be taken cautiously given the low KMO value.

**Table 3:** Factor analysis of the CB measures (Study 1).

**Cognitive bias**	**Factor 1**	**Factor 2**
1. Framing bias	1.00	0.00
2. Hindsight bias	0.00	0.53
3. Overconfidence bias	0.18	0.03
4. Anchoring bias	0.10	0.35
5. Outcome bias	0.02	0.36
6. Base rate neglect	−0.16	0.23
7. Sunk cost fallacy	−0.04	−0.02
8. Belief bias	−0.09	0.34
Eigen values	1.61	1.19
Variance explained	13%	9%

To sum up, we found lower internal consistency values than those reported by Bruine de Bruin et al. (2007) and Teovanović et al. (2015) except for anchoring bias—to some extent—and base rate neglect. The findings of Study 1 suggested that six of the eight measures of CB needed further investigation. While the item reduction might have impacted the reliability of the hindsight bias measure, other measures (in particular framing bias and overconfidence) required significant changes. In the case of framing, one could highlight that the scoring method of Bruine de Bruin et al. (2007) produced a more reliable measure (Cronbach's alpha = 0.50) and that the difference with the value reported by these authors (0.62) could be attributed to the reduced number of items used (4 vs. 14). As described in the Measures section, we argue however that the framing score should be calculated as the difference (rather than the absolute difference) between the mean ratings of the loss frames and the mean ratings of the gain frames, in accordance with the direction of risky-choice framing effects predicted by prospect theory (Kahneman and Tversky, 1979). Even though framing effects are smaller in within-subjects designs (Lambdin and Shaffer, 2009), the framing effect was particularly small here. We found a *d*-value of 0.23, which is lower than the value for risky frame (Cohen's *d* = 0.437) reported by Piñon and Gambara (2005) in their meta-analytic review of framing effects. In fact, using exactly the same decisions problems in the loss and gain versions might raise the likelihood that participants detect that feature (despite the two conditions being distanced from one another), leading them to be consistent in their responses, thereby reducing the effect size.

Since it is less prevalent in the literature on judgment and decision-making than the other biases, we did not further investigate the measurement of belief bias. In order to keep an overall testing time below 1 h, we splitted the set of CB to be improved into two subsequent separate studies.

### Study 2

The goal of study 2 was to improve three measures of CB: framing bias, overconfidence bias and outcome bias (Supplementary Material, Study 2).

#### Method

##### Participants

A total of 104 participants completed the experiment (37 males, 67 females). Participants were recruited by the PRISME Human Behavior Core Facility of the Institut du Cerveau in France and received 8 € for completing the test. The mean age was 32.81 (*SD* = 11.51) and the level of education was quite high as 88.46% of the participants reported a bachelor's degree at least. Participants gave their informed consent before taking part in the study.

##### Measures

###### Framing Bias.

Two changes were introduced. First, the wording of the items was slightly changed between the loss and gain versions while the objective information presented in the decision problems remained the same. For example, one version of the decision problem would be “Imagine that an autonomous vehicle out of control is rushing into a city crowd. If nothing is done, the accident will cause 120 deaths. Public authorities must choose between two interventions” while the other version would be “Imagine that a train that got out of control is about to derailed near a village. If nothing is done, the accident will cause 120 deaths. Public authorities must choose between two interventions.” In doing so, we expected participants to vary more their responses between the two conditions, leaving more room for the framing bias. Second, we raised the number of items (8 pairs). Items were adapted from the existing framing literature (Fischhoff, 1983; Bazerman, 1984; Fagley and Kruger, 1986; Svenson and Benson, 1993; Highhouse and Paese, 1996), one of them is used in Bruine de Bruin et al. (2007).

###### Overconfidence Bias.

As scores to a general knowledge test showed poor reliability, we used a task more likely to provide reliable accuracy scores. We selected two cognitive tasks of the International Cognitive Ability Resource (Condon and Revelle, 2014), the Matrix Reasoning and Verbal Reasoning tasks as scores in those tasks are quite reliable (Cronbach's alpha = 0.68 and 0.76, respectively). Participants indicated their confidence on a scale ranging from 0 to 100%, so that the potential range of overconfidence scores was −100 to 100.

###### Outcome Bias.

The quite poor internal consistency found in Study 1 suggested that it was primarily a matter of number of items. Accordingly, we raised the number of items up to 24. The same items as in Study 1 were used; seven of the remaining items added were drawn from the existing literature (Aczel et al., 2015; Teovanović et al., 2015) and seven new items were created.

The tasks were administered in the following order: (1) gain version items of the framing task, (2) overconfidence bias (Matrix Reasoning task then Verbal Reasoning task), (3) outcome bias, (4) loss version items of the framing task.

#### Results

The findings obtained for the three measures were satisfactory (see Table 1). The measure of framing bias produced a large effect size (*M* = 0.94, Cohen's *d* = 1.05), suggesting that participants were more prone to vary their responses between the loss and gain versions of the decision problems. The measure reached a satisfactory level of internal consistency (Cronbach's alpha = 0.74) while the value obtained with the scoring method of Bruine de Bruin et al. (2007) was 0.81. Regarding the measurement of overconfidence, scores to the tasks showed the expected level of reliability (Cronbach's alpha = 0.77 for scores to the Matrix Reasoning task, 0.81 for scores to the Verbal Reasoning task, and 0.88 for scores to the two tasks combined). Overconfidence scores reached a high level of internal consistency when the two tasks were combined (Cronbach's alpha = 0.81). The reliability was lower when considering each task separately (Cronbach's alpha = 0.69 for the Matrix Reasoning task and 0.62 for the Verbal Reasoning task). Finally, the measure of outcome bias produced a large effect size (*M* = 0.77, Cohen's *d* = 0.95) and scores showed a high level of internal consistency (Cronbach's alpha = 0.85). Correlations between the three CB measures were around 0, and none was statistically significant (see Table 2). Accordingly, no factor analysis of the measures was carried out.

The results of Study 2 confirmed that framing bias and overconfidence bias can be reliably measured but under certain task conditions. For the measurement of framing bias, in addition to being separated in the test, the loss and gain versions of the items should be worded differently so that participants are less likely to detect their similarity, leaving more room for the framing effect to occur. Regarding the measurement of overconfidence, the task used must provide reliable accuracy scores. Accordingly, cognitive tasks should be preferred to general knowledge tests. Noteworthy, the results also revealed that our measurement procedure of the outcome bias (using different items for the positive and negative outcome conditions), which does not require the two outcome conditions to be temporally separated, provides reliable scores. Note that regarding overconfidence bias and outcome bias, a reduced number of items might be used without hurting much reliability.

### Study 3

#### Method

The goal of Study 3 was to improve the measurement of hindsight bias and sunk cost fallacy (with respect to the results of Study 1), and test a measure of confirmation bias (Supplementary Material, Study 3).

##### Participants

The participants were 102 unpaid undergraduate students (19 males, 83 females) who attended first-year introductory course in differential psychology at the University of Lorraine (France). The mean age was 18.47 (*SD* = 1.38). Participants gave their informed consent before taking part in the study.

##### Measures

###### Hindsight Bias.

Two changes were introduced with respect to Study 1: the number of items was raised up to 15 (the set of words added were also new) and the two phases of the task were further separated in the test in order to reduce the likelihood that during the recall participants accurately remembered the confidence in their responses in the first phase.

###### Sunk Cost Fallacy.

As it was unlikely that the low internal consistency found in Study 1 was entirely due to the reduction of the number of items, we simply changed the items. Five items were adapted from the material used in the sunk-cost literature (Arkes and Blumer, 1985; Bornstein and Chapman, 1995; e.g., “After a large meal at the restaurant, you order a good dessert. After a few bites, you realize that you are no longer hungry. Would you rather keep eating or stop eating?”).

###### Confirmation Bias.

Confirmation bias is the tendency to search for, to interpret, to favor, and to recall information that confirms or supports one's prior personal beliefs (Nickerson, 1998). Despite its prevalence in the judgement and decision-making literature (e.g., Lilienfeld et al., 2009), our review revealed that no study has reported a reliable—objective—measure of this bias. We addressed that issue following the exploratory work of Gertner et al. (2016). As highlighted in the definition of Nickerson (1998) and outlined in the work of Gertner et al., confirmation bias has several aspects (e.g., seeking evidence consistent with a previously-made decision or with a previously formulated hypothesis; assigning greater weight to evidence that is consistent with a prior hypothesis or belief than to disconfirming evidence). We aimed to measure the “weighting of evidence” facet relying upon the task used by Snyder and Swann (1978). In this task, participants are provided with a hypothesis regarding an interviewee's personality (e.g., the candidate is extroverted) and then select which questions to ask to the interviewee to evaluate the hypothesis. Some of the questions assume that the candidate has the personality trait while other questions assume that the candidate has not the personality trait. The rationale is that participants prone to confirmation bias will favor the first category of questions. We used four items; in each one, participants were asked to select among a set of 20 questions eight that could test the hypothesis that the interviewee had a given personality trait (e.g., extroverted). The set of 20 questions included eight questions assuming that the candidate had the personality trait (e.g., what events make you feel popular with people?), eight questions assuming that the candidate had not the personality trait (e.g., what things do you dislike about loud parties?), and four neutral questions (e.g., what are some of your favorite books?). The confirmation bias score was the percentage of questions assuming that the candidate had the personality trait chosen by the participant. Each item involved a particular personality trait (agreeableness, conscientiousness, emotional stability, extroversion). The material (list of questions) was drawn from Sackett (1979, 1982).

The tasks were administered in the following order: (1) the first phase of the hindsight task, (2) sunk cost fallacy, (3), confirmation bias, (4) the second phase of the hindsight task (recall). After completing the test, participants were given feedback on the study.

#### Results

As in Study 1, we were unable to obtain a satisfactory level of internal consistency for the sunk cost fallacy measure (Cronbach's alpha = 0.08) despite a large effect size (*M* = 3.61, Cohen's *d* = 2.99) similar to that observed in Study 1 (see Table 1). The measure of hindsight bias reached a near-acceptable level of internal consistency (Cronbach's alpha = 0.62), confirming that the value found in Study 1 was due to item reduction. Note that the value obtained with the scoring method of Teovanović et al. (2015) was 0.50 (a value closed to that obtained in Study 1), which is still below that reported by these authors (0.66). Finally, the measure of confirmation bias showed positive results. Results of the Shapiro-Wilk test showed that scores were normally distributed (*W* = 0.99, *p* = 0.47). The effect size was substantial (*M* = 51.67, Cohen's *d* = 3.64) and the range of observed scores was extended (20.83–87.5) thus indicating a good discriminative ability. The measure also reached a near-acceptable level of internal consistency (Cronbach's alpha = 0.68) with only four items. Correlations between the three CB measures were around 0, and none was statistically significant (see Table 2). Accordingly, no factor analysis of the measures was carried out.

## Discussion

The present study addressed the issue of the measurement of individual differences in CB. We first reviewed the corresponding psychometric literature, which is sparse. Indeed, most research in decision-making that included measures of individual differences in CB used composite scores based on various, single-item, and tasks. Such aggregated scores showed poor reliability (e.g., Toplak et al., 2011; Aczel et al., 2015). Two valuable studies aimed at improving scale reliability using multi-item tasks (Bruine de Bruin et al., 2007; Teovanović et al., 2015). Taken together, these two studies indicate that a set of eight independent CB measures—forming an inventory—are currently available and ready to be used by other researchers.

We secondly aimed to replicate, improve and extend this inventory. Through three studies, we were able to obtain reliable measures for six of the eight CB identified. For three measures, this required adjustments regarding the scoring method (framing bias and hindsight bias) or the task itself (framing bias and overconfidence bias). Moreover, we provided two main improvements. First, we improved the measurement of the framing bias reported by Bruine de Bruin et al. (2007) and the measurement of the hindsight bias and outcome bias reported by Teovanović et al. (2015). For these three CB, we suggest that our adjusted version and/or scoring method should be used. For the other CB, the original measure as described in Bruine de Bruin et al. (2007) or Teovanović et al. (2015) can be used. Second, we provided evidence for a reliable measurement of the confirmation bias using only four items. That measure, however, is relative to only one aspects of the bias (weighting of evidence) and thus calls for further investigation. In particular, avenues for future research involve developing reliable measures of other aspects (e.g., seeking evidence) and investigating the correlations between these measures to see if a general factor reflecting the bias can be extracted (Gertner et al., 2016).

The present study has several limitations. First, one could highlight that our samples across studies were quite small, and that samples in Study 1 and 3 were not gender-balanced (females being over-represented in comparison to males) and particularly young (mean age between 18 and 19). In fact, gender and age are known to influence risk-taking and decision-making. Regarding gender, women are more risk-averse than men generally (e.g., Lauriola and Levin, 2001). That might shed light on the absence of framing effect in Study 1 as females may have avoided the risky-choice option both in the gain and the loss conditions. Regarding age, studies have generally found that young adults are less risk-averse than adults (e.g., Boyer, 2006) and such differences might expand to decision-making in general. To explore this issue, we tested the effect of gender and age on the different bias scores in Study 2 (the standard deviation of age was too small in Study 1 and 3). The effect of gender on framing scores was not significant, but the effect on overconfidence scores was significant [*F*_(2,100)_ = 3.47, *p* < 0.05], males (*M* = 9.61) being more overconfident than females (*M* = 4.16). Gender had also a significant effect on outcome bias scores [*F*_(2,100)_ = 3.82, *p* < 0.05], females (*M* = 1.01) being more prone to this bias than males (*M* = 0.34). On the other hand, age had no significant effect on framing scores, but it had a significant effect on overconfidence scores [*F*_(1,100)_ = 8.42, *p* < 0.01] and outcome bias scores [*F*_(1,100)_ = 8.47, *p* < 0.01]. In both cases, age was positively correlated with the bias score. This suggests that effect sizes might be larger in adult samples compared to young adult ones, so that measures of CB may be less reliable in the latter. The mean age of samples in Study 1 and 3 was actually below that in the studies of Bruine de Bruin et al. (2007) (*M* = 47.7) and Teovanović et al. (2015) (*M* = 19.83).

Second, in most measures, the reduction of the number of items was paralleled by a change in the set of items (and in the task itself in the case of outcome bias), so that the effects of both manipulations were confounded. In fact, this would be problematic in cases in which the internal consistency found is lower than the original value. This was the case only for the sunk cost fallacy and belief bias. In the case of belief bias, as the items used were drawn from the original measure, the low internal consistency found could only originate in the item reduction. Regarding the measure of sunk cost fallacy, the low values obtained could be attributed either to the reduced number of items and/or to the change of the set of items. However, both possibilities seem unlikely as the cut in the number of items was small (5 vs. 10 and 8), and the change of the set of items between Study 1 and Study 3 did not result in any improvement of reliability. Further research is needed to further clarify why this measure seems problematic, using the same number of items as in the original measure (8 or 10). More generally, future studies on CB measurement should follow the guideline of first validating the measurement procedure (task, items, and scoring) and then determining the minimum number of items.

Third, while we examined the reliability of CB measures, we focused only on internal consistency and did not consider test-retest reliability. Future studies will have to address this issue to fully evidence the reliability of these measures.

Our review and findings highlight that the measurement of individual differences in CB is still in its infancy, in several respects. First, the current measures of CB still require improvement. In particular, we were unable to obtain reliable measures for the sunk cost fallacy and belief bias despite replicating the original procedure.

Second, measures of key CB are still lacking. Despite their prevalence in the decision-making literature, measures of individual differences in confirmation bias and availability bias or not available. This is a clear sign that individual differences have been neglected in this field of research. In particular, measures relative to CB in probabilistic thinking (e.g., gambler's fallacy, regression to the mean, covariation detection) are lacking. To address that issue, future research could leverage previous studies that used various (single-item) tasks of probabilistic thinking (e.g., Toplak et al., 2007; see also Aczel et al., 2015). Furthermore, while we obtained positive results regarding the measurement of confirmation bias, we just started tackling that issue and much research remains to be done to reach a full measure of this bias.

Third, the eight measures of CB considered here are *generic* measures, using non-contextualized items. Such measures are relevant for research with the purpose of describing general aspects of decision-making (e.g., Del Missier et al., 2020b). They may also be useful for research on debiasing, which involves pretest/post-test designs (e.g., Morewedge et al., 2015). In such studies, relying upon a reliable measurement of individual differences in CB would allow to check for differential effects of the debiasing intervention. For instance, the intervention might work on individuals moderately susceptible to the bias targeted while having no or even adverse effects on individuals highly susceptible to it. The same reasoning holds in the context of behavioral public policy: policymakers should take full account of individual differences as any single intervention may have varying effects on different people (Rachlinski, 2006). On the other hand, research on individual differences in decision-making in experts or professionals might require *specific* measures adapted to the context in which a particular decision is made (e.g., management decision, diagnostic decision, and sentencing decision). This work was initiated in medicine for example with the Inventory of Cognitive Biases in Medicine (ICBM; Hershberger et al., 1994), an instrument which aims to measure ten CB in doctors (e.g., insensitivity to prior probability, insensitivity to sample size) through 22 medical scenarios with forced choice responses. While the ICBM failed to achieve satisfactory psychometric properties (Sladek et al., 2008), this work illustrates the need to further develop contextualized measures.

The measurement of individual differences in CB is still at the stage of establishing reliable measures. Once satisfactory levels of reliability are reached, the next step is to investigate the validity of the measures. An important avenue of research is to compare generic and specific measures of CB with respect to criterion validity. For instance, using a version of the Wason task adapted to the context of arbitration, Helm et al. (2016) reported that arbitrators are prone to the confirmation bias. Interestingly, they hypothesized that “The arbitrators' vulnerability to the confirmation bias might increase the cost and duration of arbitration” (p. 685). Do arbitrators that are the most prone to the confirmation bias actually have costly and longer arbitration? Such criterion-related studies, which are currently lacking (but see Parker and Fischhoff, 2005), would be critical to further establishing the relevance of the measurement of individual differences in CB.

## Data Availability Statement

The datasets presented in this study can be found in online repositories. The names of the repository/repositories and accession number(s) can be found at: https://osf.io/wfums/.

## Ethics Statement

The studies involving human participants were reviewed and approved by Laboratoire Lorrain de Psychologie et Neurosciences de la Dynamique des Comportements (2LPN, EA 7489). The patients/participants provided their written informed consent to participate in this study.

## Author Contributions

VB: study conception and design, data collection, analysis and interpretation of results, and manuscript preparation.

## Conflict of Interest

The author declares that the research was conducted in the absence of any commercial or financial relationships that could be construed as a potential conflict of interest.

## Footnotes

^1^For instance, the Decision Making Individual Differences Inventory (DMIDI; http://www.sjdm.org/dmidi/), a public resource that categorizes and describes the most common individual difference measures used in judgment and decision-making research (Appelt et al., 2011), does not include any specific measure of CB with the partial exception of the Adult Decision-Making Competence (A-DMC; Bruine de Bruin et al., 2007).

^2^Among the seven tasks of the A-DMC, we retained only the three measuring constructs identified as CB *per se* (resistance to framing, overconfidence, and resistance to sunk costs).
