# Supplementary material for: The Measurement of Individual Differences in Cognitive Biases: A Review and Improvement
Source: Front Psychol. 2021 Feb 18;12:630177. doi: 10.3389/fpsyg.2021.630177 (PMC7930832; doi:10.3389/fpsyg.2021.630177)
Supplement: Supplementary file 1 [file Data_Sheet_1.docx]

Supplementary Material

**The measurement of individual differences in cognitive biases: A review and improvement**

**Vincent Berthet**

**Correspondence:** [vincent.berthet@univ-lorraine.fr](mailto:vincent.berthet@univ-lorraine.fr)

We report here all materials used in the three studies. All items were originally presented in French, we report here their English version or translation.

# **1. Study 1**

## **1.1 Framing bias**

*Gain frames*

1. Imagine that France is preparing for the outbreak of an unusual disease, which is expected to kill 600 people. Two alternative programs to combat the disease have been proposed. Assume that the exact scientific estimates of the consequences of the programs are as follows:

If program A is adopted, 200 people will be saved.

If program B is adopted, there is a 33% chance that 600 people will be saved, and a 67% chance that no people will be saved.

Which option would you choose? [Tversky and Kahneman (1981)]

2. Because of changes in tax laws, you may get back as much as 1200 euros in income tax. Your accountant has been exploring alternative ways to take advantage of this situation. He has developed two plans:

If plan A is adopted, you will get back 400 euros of the possible 1200 euros.

If plan B is adopted, you have a 33% chance of getting back all 1200 euros, and a 67% chance of getting back no money.

Which option would you choose? [Highhouse and Paese (1996)]

3. Imagine that three years ago you bought a house. Six months ago, your home was appraised for 36 000 euros more than you paid for it. Now your employer is transferring you to Lyon, and you must sell you house. Unfortunately, the real estate market has declined in recent months and the best offer you have is only 12 000 euros more than you paid for it. You cannot wait for the market to improve; you must sell now. You contacted a real estate broker who has suggested two possible options:

Plan A: Sell your house not for the current best offer and save 12 000 euros of the appreciation.

Plan B: Sell your house at an auction. There is a 1/3 chance you will save all of the 36 000 euros appreciation, and a 2/3 chance that you will save none of the 36 000 euros appreciation.

Which option would you choose? [Fagley and Miller (1997)]

4. Imagine an African village in which the children have been severely food poisoned. If nothing is done, 120 children are estimated to die. There are two alternative programs for curing the children:

The program A will save 30 children.

The program B provides a 25% chance that everybody is saved and 75% that nobody will be saved.

Which option would you choose? [Svenson and Benson (1993)]

*Loss frames*

1. Imagine that France is preparing for the outbreak of an unusual disease, which is expected to kill 600 people. Two alternative programs to combat the disease have been proposed. Assume that the exact scientific estimates of the consequences of the programs are as follows:

If program A is adopted, 400 people will die.

If program B is adopted, there is a 33% chance that nobody will die, and a 67% chance that 600 people will die.

Which option would you choose?

2. Because of changes in tax laws, you may get back as much as 1200 euros in income tax. Your accountant has been exploring alternative ways to take advantage of this situation. He has developed two plans:

If plan A is adopted, you will lose 800 euros of the possible 1200 euros.

If plan B is adopted, you have a 33% chance of losing none of the money, and a 67% chance of losing all 1200 euros.

Which option would you choose?

3. Imagine that three years ago you bought a house. Six months ago, your home was appraised for 36 000 euros more than you paid for it. Now your employer is transferring you to Lyon, and you must sell you house. Unfortunately, the real estate market has declined in recent months and the best offer you have is only 12 000 euros more than you paid for it. You cannot wait for the market to improve; you must sell now. You contacted a real estate broker who has suggested two possible options:

Plan A: Sell your house not for the current best offer and lose 24 000 euros of the appreciation.

Plan B: Sell your house at an auction. There is a 1/3 chance you will lose none of the 36 000 euros appreciation, and a 2/3 chance that you will lose all of the appreciation.

Which option would you choose?

4. Imagine an African village in which the children have been severely food poisoned. If nothing is done, 120 children are estimated to die. There are two alternative programs for curing the children:

The program A will leave 90 children to die.

The program B provides a 25% chance that nobody dies and 75% chance that everybody dies.

Which option would you choose?

## **1.2 Hindsight bias**

The underlined word is the odd one out. For items 6, 7, 8 and 9 we also indicate the French words as the rule to find the odd one out is specific to the French language.

1. Carbon, Copper, Iron, Aluminum

2. Cheese, Butter, Milk, Yogurt

3. Arrow, Shield, Dagger, Spear

4. Earth, Moon, Pluto, March

5. November, August, December, January

6. Fiançailles (Engagement), Funérailles (Funeral), Noces (Wedding), Ténèbres (Darkness)

7. Autobus (Bus), Automobile (Automobile), Avion (Plane), Hélicoptère (Helicopter)

8. Citronnelle (Lemongrass), Coupelle (Cup), Poutrelle (Beam), Ruelle (Alley)

9. Demain (Tomorrow), Dehors (Outside), Déjà (Already), Aucun (None)

10. Hatred, Fear, Greed, Anger

## **1.3 Overconfidence bias**

1. Which famous dictator ruled the USSR from the mid-1920s to 1953?

Stalin/Lenin

2. Which filmmaker directed “Parle avec elle” and “Volver”?

Guillermo del Toro/Pedro Almodovar

3. Is the penguin a bird?

Yes/No

4. In which Italian city is the action of Shakespeare’s play “Romeo and Juliet” located?

Venice/Verona

5. Who called himself “France’s first cop”?

Charles de Gaulle/Georges Clemenceau

6. The Russian flag is white, blue and …?

Red/Black

7. To which composer do we owe the soundtracks of films like “Once Upon a Time in America”, “A Fistful of Dollars”, or “The Sicilian Clan”?

Ennio Morricone/Hans Zimmer

8. In which city is the Colosseum (the Flavian Amphitheater) located?

Barcelona/Rome

9. Which famous writer begins his plea in favor of Captain Dreyfus with “I accuse ...”?

Emile Zola/Victor Hugo

10. Which of these films has not been directed by Steven Spielberg?

Gladiator/ Saving Private Ryan

11. Who composed “Ode to Joy”, the anthem of the European Union?

Beethoven/Wagner

12. Which Spanish writer is the author of Don Quixote?

Miguel de Cervantes/Tirso de Molina

13. Which is the correct spelling?

Choux-fleurs/Choux-fleur

14. In what year did Neil Armstrong said “That’s one small step for man, one giant leap for mankind”?

1968/1969

15. Which of these cities is the most populous (including the suburbs)?

Paris/Madrid

16. We say that a horse:

Cockroach/Neighs

17. Which of the following politicians took power in Cuba in 1959?

Fidel Castro/Hugo Chavez

18. Of which writer is the “Human Comedy” the great work?

Honoré de Balzac/Emile Zola

19. Who is the most successful athlete in the history of the Olympic Games?

Carl Lewis/Michael Phelps

20. What is the largest state in the world?

China/Russia

21. Which painter is the main representative of cubism?

Picasso/Rubens

22. What is the capital of Morocco?

Casablanca/Rabat

23. What city is Chelsea Football Club in?

Manchester/London

24. “God and my right”: What country is this the motto?

United Kingdom/Belgium

25. What group was Jim Morrison the singer of?

Deep Purple/The Doors

## **1.4 Anchoring bias**

1. The heat record in the city of Paris (in °C).

2. The age of Napoleon Bonaparte when he died.

3. The average monthly salary for the first job of students graduating from a business school in France.

4. The average number of births per day in France.

5. The weight of Julius Caesar.

6. The age of Albert Camus when he wrote his first novel.

7. The average length of a dinner at home in France (in minutes).

8. The year Raymond Poincaré was elected President of the French Republic.

9. The boiling point of water at the top of Everest (in °C).

10. The number of states in the United States in 1840.

11. The number of African states in the United Nations.

12. The gestation period of the African elephant (in months).

## **1.5 Outcome bias**

*Decisions with a positive outcome*

1. A researcher at a pharmaceutical company is responsible for carrying out a study testing the effectiveness of a molecule for the treatment of an allergy. He is due to release the study report soon and the results are inconclusive: the effect of the molecule is not apparent. Looking at the statistical analyzes, he found that the data for 4 patients had been removed from the analyzes due to anomalies. He realizes that by including these patients, the effect of the molecule becomes apparent. He decided to include these patients. The molecule has been brought to the market and is found to be effective and has no side effects.

Evaluate the researcher’s decision to include the data from the 4 patients. [Adapted from Gino, Moore, and Bazerman (2009)]

2. Celine has a college exam in two days. A friend of hers invited her to a party yesterday. She decided to go. She had a great time and stayed with her friend until the early hours of the morning. The next day, she revised most of the day. She passed her exam.

Evaluate Celine’s decision to go to the party. [Adapted from Teovanović et al. (2015)]

3. There has been an accident in a mine and the miners are stranded. In an emergency, the public authorities decided to dig a tunnel to pick up the trapped miners. Experts estimated that there was a 40% chance that the operation would work and a 60% chance that the situation would get dramatically worse. The operation was successful and all the miners were rescued.

Evaluate the decision of public authorities to dig a tunnel. [Adapted from Teovanović et al. (2015)]

4. Marc is a lawyer and his client was sentenced to 5 years in prison by the Assize Court. Marc estimates that there is a 7 in 10 chance that the sentence will be more severe if the case is retried by the court of appeal. He still appeals the judgment, and his client is released on appeal.

Evaluate Marc’s decision to appeal. [New item]

5. A group of four friends are planning to go on vacation to Cannes for a week and one of them, Thierry, is in charge of booking an apartment. He targeted two apartments, a first in the city center at 300 euros per night and a second off-center at 240 euros per night. When he submits these two possibilities to his friends, they point out to him that the photos of the second apartment cast doubt on its quality. Thierry still chooses the second apartment. The four friends had an excellent stay in Cannes and they were fully satisfied with the apartment.

Evaluate Thierry’s decision to choose the second apartment. [New item]

*Decisions with a negative outcome*

1. A 55-year-old man had a heart condition. He had to stop working because of chest pain. He enjoyed his work and did not want to stop. His pain also interfered with other things, such as travel and recreation. A type of bypass operation would relieve his pain and increase his life expectancy from age 65 to age 70. However, 8% of the people who have this operation die from the operation itself. His physician decided to go ahead with the operation. The operation failed and the man died.

Evaluate the physician’s decision to go ahead with the operation. [Baron and Hershey (1988)]

2. A biotechnology company is considering the development of a new, innovative technology. If the new technology is successfully introduced to the market, the investment will have a high rate of return. However, experts consider the investment very risky, because the company has to take out a large loan to cover development costs. According to the analysts, there is a 10% chance that the project will fail and the whole company will go bankrupt. The management decided to invest in the development, but it was unsuccessful and the company went bankrupt.

Evaluate the company’s decision to invest in the project. [Aczel et al. (2015)]

3. Mathieu wants to buy a pair of shoes but he cannot find the model he is looking for in the store. He decides to buy it online. The size indicated on the site is the American size, he chooses the size equivalent to the European size. When he receives the package, Mathieu realizes that the size is too big and has to send the package back.

Evaluate Mathieu’s decision to order his pair of shoes online. [Adapted from Teovanović et al. (2015)]

4. You are late for a college exam. Being stuck in traffic, you decide to walk to college as quickly as possible. But you arrive late and you are not allowed to take the exam.

Evaluate your decision to walk to college. [Teovanović et al. (2015)]

5. One Friday night, Axel goes to a Japanese restaurant with some friends. Looking at the menu, he must decide between ordering his favorite dish or a new dish that looks tempting. He finally chooses the new dish. It turns out that Axel didn’t like this dish and didn’t even finish it.

Evaluate Axel’s decision to choose the new dish over his favorite dish. [New item]

## **1.6 Base rate neglect**

1. In a study 1000 people were tested. Among the participants there were 4 men and 996 women. Dominique is a randomly chosen participant of this study. Dominique is 23 years old and is finishing a degree in engineering. On Friday nights, Dominique likes to go out cruising with friends while listening to loud music and drinking beer.

What is the probability that Dominique is a man? [De Neys & Glumicic (2008)]

2. In a study 1000 people were tested. Among the participants there were 997 nurses and 3 doctors. Paul is a randomly chosen participant of this study. Paul is 34 years old. He lives in a beautiful home in a posh suburb. He is well spoken and very interested in politics. He invests a lot of time in his career.

What is the probability that Paul is a doctor? [De Neys & Glumicic (2008)]

3. In the Blaise Pascal high school in Rouen, 10% of students in their last year access to preparatory classes for grandes écoles. Sophie is in her final year at this high school, she regularly obtains grades above 16 and she is appreciated by her teachers.

What is the probability that Sophie integrates a preparatory class? [New item]

4. In Greece, 15% of women marry before the age of 25. Katerina is a 23-year-old Greek woman who has been in a serious relationship for three years.

What is the probability that Katerina will marry before she is 25? [New item]

## **1.7 Sunk cost fallacy**

1. Imagine that you are the head of a car manufacturing company, and you have accepted a project to build a new model. The cost of the project is 80 million euros and the investment amount to date is 70 million euros. Your competitor is about to release a car that uses less fuel than yours, is greener, and at a much lower price than yours.

Would you be more likely to abandon your project or invest the last 10 million euros? [Adapted from Arkes and Blumer (1985)]

2. A few months ago, you bought a ticket to attend a show that is an hour drive from your home. On the day of the show, you feel a little sick and a heavy rain will make your trip unpleasant.

Would you be more likely to stay at home or go to the show? [New item]

3. You bought two tickets to watch a soccer game with your son. A few days before the game, your son’s favorite player gets injured and he is no longer interested in going to the game.

Would you be more likely to do something else or go to the game? [Adapted from Bornstein and Chapman (1995)]

4. It is the last day of your summer vacation. For that day, you have previously paid for a boat-excursion to a nearby island. Just before, you are offered a free activity: one hour of diving with an instructor.

Would you be more likely to choose the diving or the boat trip? [Teovanović et al. (2015)]

5. You have worked for 6 months on a business creation project and you paid a consultant 1000 euros to assess the quality of your project. She tells you that the project is not viable because the market is in decline.

Would you be more likely to stop your project or continue it? [New item]

## **1.8 Belief bias**

Items are from Teovanović et al. (2015).

Pair 1

| Consistent item (valid and believable) | Inconsistent item (valid and unbelievable) |
| --- | --- |
| All humans are mortal. I am a human. I am mortal. | All mammals walk. Whales are mammals.  Whales walk. |

Pair 2

| Consistent item (invalid and unbelievable) | Inconsistent item (invalid and believable) |
| --- | --- |
| Everything wooden is fuel. Gas is not wooden. Gas is not fuel. | All the African countries are poor. Switzerland is not an African country. Switzerland is not poor country. |

Pair 3

| Consistent item (invalid and unbelievable) | Inconsistent item (invalid and believable) |
| --- | --- |
| All trolleybuses use power. Boilers use power. Boilers are trolleybuses. | All living beings need water. Roses need water. Roses are living beings. |

Pair 4

| Consistent item (valid and believable) | Inconsistent item (valid and unbelievable) |
| --- | --- |
| All fruits are edible. Cigarettes are not edible. Cigarettes are not fruits. | All birds can fly. Ostrich cannot fly. Ostrich is not a bird. |

# **2. Study 2**

## **2.1 Framing bias**

*Gain frames*

1. Imagine that due to a restructuring plan, the Renault group will potentially have to lay off 6000 employees in France. The management is considering two plans to avoid such a situation:

If plan A is adopted, 2000 jobs will be saved.

If plan B is adopted, there is a 1/3 chance of saving the 6000 jobs and a 2/3 chance that no job will be saved.

Which option would you choose? [Adapted from Bazerman (1984)]

2. Imagine a storm approaching a city of 150 000 people. If nothing is done, 12 000 homes could be degraded. Public authorities must choose between two interventions:

If plan A is adopted, 3000 homes will be saved.

If plan B is adopted, there is a 25% chance of saving all 12 000 homes and a 75% chance that no home will be saved.

Which option would you choose? [Adapted from Fischhoff (1983)]

3. Imagine that after a serious traffic accident, 60 people are stranded in a tunnel. Public authorities must choose between two interventions:

If plan A is adopted, 20 people will be saved.

If plan B is adopted, there is a 1/3 chance of saving 60 people and a 2/3 chance of not saving anyone.

Which option would you choose? [Adapted from Wang (1996)]

4. Imagine that you have just spilled liquid on your external hard drive. 12 GB of personal data is at stake. You must quickly choose between two options:

If you choose option A, you will save 3 GB of data.

If you choose option B, there is a 1/4 chance of saving your 12 GB of data and a 3/4 chance that you will save no data.

Which option would you choose? [Adapted from Svenson and Benson (1993)]

5. Imagine that a group of 20 patients with the same heart disease have to undergo surgery. Doctors must choose between two interventions:

If intervention A is chosen, 6 patients will be cured.

If intervention B is chosen, there is a 30% chance that all 20 patients will be cured and a 70% chance that none will be cured.

Which option would you choose? [Svenson and Benson (1993)]

6. Imagine that an autonomous car out of control is rushing into a city crowd. If nothing is done, the accident will cause 120 deaths. Public authorities must choose between two interventions:

If intervention A is chosen, 30 people will be saved.

If intervention B is chosen, there is a 25% chance of saving 120 people and a 75% chance that no one will be saved.

Which option would you choose? [Adapted from Svenson and Benson (1993)]

7. Imagine that France is preparing for the outbreak of an unusual Asian disease, which is expected to kill 600 people. Two alternative programs to combat the disease have been proposed. Assume that the exact scientific estimates of the consequences of the programs are as follows:

If program A is adopted, 200 people will be saved.

If program B is adopted, there is a 1/3 chance that 600 people will be saved, and a 2/3 chance that no people will be saved.

Which option would you choose? [Tversky and Kahneman (1981)]

8. Imagine that signs of contamination of drinking water with tritium (an isotope released from nuclear power stations) have been reported. If nothing is done, the 5000 inhabitants of the nearest town could be poisoned. Public authorities must choose between two interventions:

If intervention A is chosen, 1500 residents will be saved from poisoning.

If intervention B is chosen, there is a 30% chance of saving all 5000 inhabitants and a 70% chance that no people will be saved.

Which option would you choose? [Adapted from Fagley and Kruger (1986)]

*Loss frames*

1. Imagine that due to a restructuring plan, a company in the Metallurgy sector will potentially have to lay off 6000 employees in France. The management is considering two plans to avoid such a situation:

If plan A is adopted, 4000 employees will be laid off.

If plan B is adopted, there is a 1/3 chance of not firing any employee and a 2/3 chance of firing the 6000.

Which option would you choose?

2. Imagine a tsunami approaching a city of 150 000 people. If nothing is done, 12 000 homes could be degraded. Public authorities must choose between two interventions:

If plan A is adopted, 9000 homes will be degraded.

If plan B is adopted, there is a 25% chance that no home will be affected and a 75% chance that all 12 000 homes will be affected.

Which option would you choose?

3. Imagine that after a fire in a college, 60 students are stranded in a classroom. Firefighters must choose between two interventions:

If plan A is adopted, 40 students will die.

If plan B is adopted, there is a 1/3 chance that no student will die and a 2/3 chance that all 60 students will die.

Which option would you choose?

4. Imagine that your laptop is attacked by a computer virus. 12 GB of personal data is at stake. You must quickly choose between two options:

If you choose option A, you will lose 9 GB of data.

If you choose option B, there is a 1/4 chance of not losing any data and a 3/4 chance of losing all data.

Which option would you choose?

5. Imagine that a group of 20 patients with the same cancer have to undergo surgery. Doctors must choose between two interventions:

If intervention A is chosen, 14 patients will die.

If intervention B is chosen, there is a 30% chance that no patient will die and a 70% chance that all 20 patients will die.

Which option would you choose?

6. Imagine that a train out of control is about to derail near a village. If nothing is done, the accident will cause 120 deaths. Public authorities must choose between two interventions:

If intervention A is chosen, 90 people will die.

If intervention B is chosen, there is a 25% chance that no one will die and a 75% chance that 120 people will die.

Which option would you choose?

7. Imagine that France is preparing to face an epidemic wave that is expected to cause 600 deaths. Medical specialists have put forward two programs to deal with this situation. According to them, the effects of these programs are as follows:

If program A is adopted, 400 people will die.

If program B is adopted, there is a 1/3 chance that nobody will die, and a 2/3 chance that 600 people will die.

Which option would you choose?

8. Imagine that there are signs of a radioactive gas leak at a nuclear power station. If nothing is done, the 5000 inhabitants of a nearby municipality could be poisoned. Public authorities must choose between two interventions:

If intervention A is chosen, 3500 inhabitants will be poisoned.

If intervention B is chosen, there is a 30% chance that no inhabitant will be affected and a 70% chance that the 5000 inhabitants will be affected.

Which option would you choose?

## **2.2 Overconfidence bias**

All items of the International Cognitive Ability Resource are available at: <https://icar-project.com/>

## **2.3 Outcome bias**

*Decisions with a positive outcome*

The first five items were those used in Study 1.

6. An acquaintance of you told you about an interesting investment opportunity in a start-up. There is a 10% chance that you will achieve a very high return on your investment and the amount of the investment is significant. You have decided to make this investment. The start-up has been successful, and you have made significant gains.

Evaluate your decision to invest in this start-up. [Adapted from Aczel et al. (2015)]

7. You are going on vacation. On your arrival at your destination, the tourist agency offers you a 20% reduction on your reservation (around 100 €) if you agree to share your accommodation with another person, whom you do not know. You accept. It turns out that you get along perfectly with your roommate and have a great vacation together.

Evaluate your decision to accept the tourist agency’s offer. [Teovanović et al. (2015)]

8. Arthur plays roulette at the casino. He bet on the right color 5 times in a row. After a few seconds of thinking, he decides to bet all his money on the next move. He wins again and doubles his stake.

Evaluate Arthur’s decision to bet all his money on his last move. [Teovanović et al. (2015)]

9. In a penalty shootout, a team has a chance to win the final of a tournament if they score the next shot. In order to surprise the opposing goalkeeper, the coach chooses a player for this shot who usually never takes a penalty. The player shoots, the ball ends its course in the net, and his team wins the final.

Evaluate the coach’s decision to choose this player. [New item]

10. Maxime is a first-year law student and is going to take an exam session. For the constitutional law exam, two subjects are considered: “Article 16 of the Constitution” and “Cohabitation under the Fifth Republic”. Maxime thinks that in the last lesson, clues given by the professor suggest that the exam will cover the subject on the article 16 of the Constitution. He chooses to revise only his courses on this topic. The exam was on the article 16 of the Constitution and Maxime was given a mark of 18/20.

Evaluate Maxime’s decision to revise only this topic. [New item]

11. Laurent wants to become a municipal councilor for his small town of Yonne. As the municipal elections approached, the two running candidates (including the incumbent mayor) offered him to be on their list. After a first mandate, it is thought that the incumbent mayor will carry out a second one. Laurent is betting that the other candidate, young and dynamic, will win and chooses accordingly to join his list. The incumbent mayor is beaten and Laurent joins the municipal council.

Evaluate Laurent’s decision to join the incumbent mayor’s competing candidate. [New item]

12. Eric went to his friend Bertrand to help him assemble a piece of furniture. At around 7:00 p.m., Bertrand told Eric that he was going to have some friends at his house tonight and offered to spend the evening with them. Eric is reluctant because he knows he tends to get bored during these evenings. He chooses to stay with his friend Bertrand. Eric had a great evening and was not bored for a second.

Evaluate Eric’s decision to spend the evening with his friend Bertrand. [New item]

*Decisions with a negative outcome*

The first five items were those used in Study 1.

6. Your friend is playing a TV game. The host of the program offers him the following choice. He can play a coin toss, in this case, if the coin lands heads, he wins 250 euros; if the coin lands on tails, he wins nothing. If he declines to play that toss, he is sure to win 100 euros. Your friend chooses the toss-up. The coin fell on tails and your friend won nothing.

Evaluate your friend’s decision to choose the coin toss. [New item]

7. At the supermarket, Clara must choose a cash register to pay for her groceries. She hesitates between one with a tail of 2 people and another with a tail of 3 people. In the tail with 2 people there is an elderly person who could potentially be slow. Clara chooses the cash register with the tail of 2 people. It turns out that the senior is very slow to pay for groceries and Clara realizes that she would have been much faster if she had chosen the checkout with the 3-person tail.

Evaluate Clara’s decision to choose the cash register with the tail of 2 people. [New item]

8. Séverine is a client advisor in a bank in a small town. To get to work, she can take either a departmental road or a faster but often more congested national road. She usually takes the departmental road which allows her to drive peacefully. One morning when she is late for an appointment with a client, Séverine chooses to take the national road. There is a traffic jam and she realizes it would have been faster if she had taken the departmental road.

Evaluate Séverine’s decision to choose the national road. [New item]

9. Two seconds before the end of a close basketball match between France and Spain where France is down 102-100, a player from the France team who entered the game at the previous time-out attempts a shot three points. His shot fails and France loses the match.

Evaluate the player’s decision to attempt that three-point shot. [Teovanović et al. (2015)]

10. You need a new washing machine. At first, you wanted to buy a model from a famous brand but found a model half the price from an unknown brand. As both models have the same warranty, the same performance, and are manufactured in the same country, you decide to buy the model from the unknown brand. It turns out that this washing machine keeps breaking down.

Evaluate your decision to buy the cheapest washing machine. [Teovanović et al. (2015)]

11. Ivan is considered to be a good writer, but so far he has only published press articles. Recently, he had an original idea as the subject of a novel. If he embarks on this project and his book finds some success with the public, it would launch his career as a writer. Otherwise, he will have wasted time and will be frustrated by the failure. Ivan decides to start writing his first novel. Ivan’s novel has gone unnoticed.

Evaluate Ivan’s decision to start writing his first novel. [Adapted from Teovanović et al. (2015)]

12. After 5 years together, Marie and Julien had separated because they quarreled too often. After 6 months of staying in touch, they started to meet again. Marie and Julien think they have each evolved. They decided to get back together. A year later, Marie and Julien separated again, this time for good.

Evaluate Marie and Julien’s decision to get back together. [Adapted from Teovanović et al. (2015)]

# **3. Study 3**

## **3.1 Hindsight bias**

The first 10 items were those used in Study 1. For items 11 and 12 we also indicate the French words as the rule to find the odd one out is specific to the French language.

11. Chaque (Each), Nombre (Number), Quatre (Four), Mille (Thousand)

12. Orgue (Organ), Délice (Delight), Amour (Love), Piano (Piano)

13. Plantation, Garden, Forest, Park

14. Athens, Tunisia, Cuba, China

15. Whale, Piranha, Trout, Shark

## **3.2 Sunk cost fallacy**

1. After a large meal at the restaurant, you order a good dessert. After a few bites, you realize that you are no longer hungry.

Would you be more likely to stop eating or to keep eating your dessert? [Adapted from Bornstein and Chapman (1995)]

2. Your daughter Anne decides to take cello lessons. After you buys a cello and pays for lessons (1200 euros for 3 months), Anne finds she is no longer interested and wants to quit.

Would you be more likely to let her stop or to push her to continue playing the cello? [Bornstein and Chapman (1995)]

3. A few months ago, you paid 350 euros for a rental for a weekend in Normandy. A few weeks later, you paid 200 euros for a rental for a weekend in Brittany. You think you will enjoy the weekend in Brittany more than the one in Normandy. At the last moment, you realize that you made a mistake in the dates: reservations fall on the same weekend! It is too late to get your payment refunded, you must choose one or the other.

Would you be more likely to choose the weekend in Brittany or the one in Normandy? [Adapted from Arkes and Blumer (1985)]

4. At your son’s request, you bought tickets to see his favorite band. The week of the concert, your son is invited to a birthday party and would rather go to the party than to the concert.

Would you be more likely to let your son go to the birthday party or to the concert? [Adapted from Bornstein and Chapman (1995)]

5. You have already spent 5000 euros to repair your old car. Shortly after the last repair it broke down again. The amount of repairs will be 1000 euros.

Would you be more likely to change your car or pay for new repairs? [New item]

## **3.3 Confirmation bias**

Items are drawn from Sackett (1979, 1982).

Item 1. Personality trait: Agreeableness

“This type of person is typically good-natured, gentle, and cooperative. They are generous, warmhearted, and do not mind giving of their time and energy. They are not prone to jealousy. They will accept blame if things go wrong, rather than putting the blame on others. They are willing to compromise and find ways of cooperating with others, even in difficult situations.”

*Questions assuming that the candidate has the personality trait*

1. Tell me about a time you held yourself publicly accountable for a mistake you made.

2. What do you do to keep yourself in good spirits?

3. What is it about working with other people that you enjoy most?

4. What do you do to keep from resenting another person who seems to be getting all the break?

5. Tell me about a situation in which you spent time listening to a person who wanted to talk about a problem, even though you had other important things to do.

6. How has willingness to compromise contributed to your success?

7. How do you maintain a positive outlook on life?

8. Tell me about a situation in which cooperating with others really paid off for you.

*Questions assuming that the candidate has not the personality trait*

1. What kinds of people seem to rub you the wrong one way?

2. Tell me about a time when your strong opinions got you in trouble?

3. Tell me about a time when your quick temper got you in trouble?

4. What most irritates you about others?

5. How does your jealousy of others usually show itself?

6. What are some ways in which you’ve been able to use others to help you achieve your own ends?

7. When was the last time when you tried to “get even” because someone did something that irritated you?

8. Describe a situation in which you are likely to be argumentative rather than cooperative.

*Neutral questions*

1. What do you see as your greatest strengths? Your greatest weaknesses?

2. What do you think are the most pressing problems facing America today?

3. What magazines do you subscribe to?

4. What would you say are the characteristics of a good teacher?

Item 2. Personality trait: Conscientiousness

“This type of person is typically responsible, orderly, and persevering. They can be depended on to accept responsibilities. They do not allow personal benefit or gain to conflict with ethical principles. They are painstaking and thorough, and see a job through in spite of difficulties or temptations. They are hardworking, strong-willed, and sometimes get overinvolved with details.”

*Questions assuming that the candidate has the personality trait*

1. Tell me about a situation where your concern for detail made it difficult to get a project done on time.

2. Why is it that others feel they can depend on you?

3. Describe a situation in which you avoided compromising your personal standards even under pressure to do so.

4. What do you do to keep yourself motivated to finish a task on which you are losing interest?

5. When was the last time you got so caught up in something you were doing that you forgot about everything else?

6. Tell me about some way in which you feel you differ from the typical college student.

7. Tell me about a situation where perseverance paid off for you.

8. How did you feel the last time you resisted the temptation to take “the easy way out”?

*Questions assuming that the candidate has not the personality trait*

1. How did you feel the last time someone depended on you to do something and you let them down?

2. How often do you cut classes?

3. When does your interest in your own personal life get in the way of getting important things done?

4. Do you think you’d do better in school if you were more organized? Why?

5. Tell me about a project you started and never finished.

6. In what kinds of situations are you willing to stretch your ethics to get something you want?

7. What was your reaction the last time you got a low grade on a paper because of typing errors, spelling mistakes, or bad grammar?

8. What are the projects you just can’t get motivated to start?

*Neutral questions*

1. How would other people describe you?

2. What do you do to keep up with current events?

3. What do you see yourself doing five years from now?

4. What movie that you saw in the last year impressed you most?

Item 3. Personality trait: Emotional stability

“This type of person is typically poised, calm, and composed. They rarely seem to get upset or nervous. They go on with what they are doing regardless of distractions. They do not lose their composure when provoked or when in an emergency situation. They perform well under pressure.”

*Questions assuming that the candidate has the personality trait*

1. Have you ever been in a situation where you kept your cool while everyone else fell apart?

2. Do people ever comment on how poised you appear when speaking in front of a group?

3. Do you see yourself as more relaxed and easygoing than other people? Why?

4. Tell me about a time when you handled a difficult situation well.

5. When was the last time something unexpected came up which threw off your plans? How did you adapt to the situation?

6. Tell me about a situation in which you felt yourself about to lose your cool. What did you do to prevent this from happening?

7. Describe a situation in which you finished a project in spite of pressure and distractions.

8. What do you do to keep calm in pressure situations?

*Questions assuming that the candidate has not the personality trait*

1. What is the greatest source of anxiety for you?

2. What sort of situations embarrass you?

3. Did you ever have any nervous habits which you have now overcome?

4. Is it ever difficult to keep your nervousness from shoving?

5. What is it about pressure situations that prevents you from doing your best work?

6. Do you find yourself snapping at other people over unimportant things?

7. What kinds of social situations make you feel ill at ease?

8. How do you show your nervousness?

*Neutral questions*

1. In a few sentences, how would you describe yourself?

2. What course did you enjoy most in college? Why?

3. What person has had the greatest influence on your life? Why?

4. Describe the ideal job.

Item 4. Personality trait: Extroversion

“Extroverts are typically outgoing, sociable, energetic, confident, talkative, and enthusiastic. Generally confident and relaxed in social situations, this type of person rarely has trouble making conversation with others. This type of person makes friends quickly and easily and is usually able to make a favorable impression on others. This type of person is usually seen by others as characteristically warm and friendly.”

*Questions assuming that the candidate has the personality trait*

1. What events make you feel popular with people?

2. What do you do to keep yourself in good spirits?

3. What social activities (e.g., clubs, groups, fraternities or sororities) have you been active in over the years?

4. What do you like about living situations in which there are always lots of people around?

5. In what social situations are you most likely to be outgoing and friendly?

6. What do you think the good and bad points of acting friendly and open?

7. In what social situations are you most likely to feel self-assured and confident in yourself?

8. What would you do if you wanted to liven things up at a party?

*Questions assuming that the candidate has not the personality trait*

1. In what situations do you wish you could be more outgoing?

2. Tell me about sometime when you felt left out from some social group. How did you handle these feelings?

3. What kind of events make you feel like being alone?

4. What factors make it hard for you to really open up to people?

5. Describe to me a type of social situation that invariably makes you feel ill at ease and awkward. What is it about such situations that makes you feel uncomfortable?

6. Think about times when your shyness in social situations has made you come across as being aloof. Give me an example.

7. What things do you dislike about loud parties?

8. Think about a time when you really wanted to talk to someone, but just couldn’t bring yourself to initiate conversation. What types of situations are most likely to make you feel this way?

*Neutral questions*

1. What activities do you really excel in?

2. What kind of charities do you like to contribute to?

3. What are some of your favorite books?

4. What are your career goals?
